# Emerging Roles of Strigolactones in Plant Responses to Stress and Development

**DOI:** 10.3389/fpls.2016.00434

**Published:** 2016-04-05

**Authors:** Amita Pandey, Manisha Sharma, Girdhar K. Pandey

**Affiliations:** Department of Plant Molecular Biology, University of DelhiNew Delhi, India

**Keywords:** plant hormones, strigolactones, abiotic stresses, biotic stresses

## Abstract

Our environment constantly undergoes changes either natural or manmade affecting growth and development of all the organisms including plants. Plants are sessile in nature and therefore to counter environmental changes such as light, temperature, nutrient and water availability, pathogen, and many others; plants have evolved intricate signaling mechanisms, composed of multiple components including several plant hormones. Research conducted in the last decade has placed Strigolactones (SLs) in the growing list of plant hormones involved in coping with environmental changes. SLs are carotenoid derivatives functioning as both endogenous and exogenous signaling molecules in response to various environmental cues. Initially, SLs were discovered as compounds that are harmful to plants due to their role as stimulants in seed germination of parasitic plants, a more beneficial role in plant growth and development was uncovered much later. SLs are required for maintaining plant architecture by regulating shoot and root growth in response to various external stimuli including arbuscular mycorrhizal fungi, light, nutrients, and temperature. Moreover, a role for SLs has also been recognized during various abiotic and biotic stress conditions making them suitable target for generating genetically engineered crop plants with improved yield. This review discusses the biosynthesis of SLs and their regulatory and physiological roles in various stress conditions. Understanding of detailed signaling mechanisms of SLs will be an important factor for designing genetically modified crops for overcoming the problem of crop loss under stressful conditions.

## Introduction

Plants are sessile in nature and have evolved an intricate signaling mechanism to sense, respond, and adapt to the continuously changing environmental conditions such as light, temperature, water, pathogens, and nutrient availability. Hormones play major roles in plant signaling and are divided into six major classes including auxins, ethylene (ET), cytokinin (CK), gibberellins (GAs), abscisic acid (ABA), and brassinosteroids (BRs). Additionally, some other molecules, categorized as plant hormones include jasmonic acid (JA), salicylic acid (SA), plant peptide hormones, polyamines (PA), nitric oxide (NO), and more recently discovered strigolactones (SLs) and karrikins (KAR; Moore, 1979; Wang and Irving, 2011; Smith and Li, 2014). In addition to higher plants, several microorganisms like bacteria, cyanobacteria, and fungi and metazoans (from sponges to mammals) produce phytohormones (Tsavkelov et al., 2006). Phytohormones are required in very low concentrations (10^-6^ to 10^-5^ mol/L) and play important roles during plant growth and development, either acting locally at the site of biosynthesis or by being transported to other plant organs. Moreover, they can act either by themselves or by interacting with other hormones generating elaborate signaling networks. A crosstalk between various PGRs is very crucial for plant growth and development to regulate tissue differentiation in response to diverse growth conditions (Hall, 1976; Evans et al., 1981; Wang and Irving, 2011).

In this review, we will discuss the emerging understanding of the mechanisms of SLs signaling in plants. SLs were identified in 1966 as a crystalline germination stimulant of parasitic weed, *Striga*, in the root exudates of cotton plants followed by elucidation of the strigol structure in 1972. Due to crop losses caused by parasitic weeds around the globe, SLs were generally considered as harmful plant secondary metabolites (Cook et al., 1972). Butler coined the term Strigolactones in Butler (1995) for a group of carotenoid derived lactone-containing compounds. SLs are exuded primarily from the roots in a wide variety of plant species including dicots, monocots, and primitive plants such as mosses, liverworts, charophyte green algae, and stoneworts (Delaux et al., 2012).

A beneficial role of SLs to plants was discovered in mycorrhizal symbiosis between plants and Glomeromycota fungi. In these association, the fungi forms arbuscular mycorrhizas with the roots of land plants. SLs regulate hyphal branching in arbuscular mycorrhizal fungus (AMF) symbiosis, which evolved around 460 million years ago and is credited for the evolution of land plants and rendering them more tolerant to abiotic and biotic stresses (Harrison, 1999; Liu et al., 2007). Besides functioning as an external stimulant, recent identification and characterization of shoot branching mutants from various plant species such as *more axillary growth 1-4* (*max1-4*) in *Arabidopsis*, *dwarf* and *high tillering dwarf* (*d*/*htd*) in rice, *decreased apical dominance 1* (*dad1*) in *Petunia hybrida*, *ramosus 1* (*rms1*) to *rms5* in *Pisum sativum* has established SLs as a phytohormone (**Table 1**; Leyser, 2009; Beveridge and Kyozuka, 2010). Detailed analysis of the mutants revealed additional roles of SLs in root growth and development, leaf shape and senescence, internode elongation, secondary growth, and drought stress responses (Brewer et al., 2013; de Saint Germain et al., 2013; Ha et al., 2014). In addition to the major roles listed above, SLs are also involved in signaling pathways in promoting seed germination in crop plants (Pepperman and Bradow, 1988) and rhizobium–legume interaction (Foo and Davies, 2011). In lower plants SLs promote rhizoid elongation in moss, liverworts, and stoneworts of which only liverworts show mycorrhizal symbiosis (Delaux et al., 2012).

**Table 1:** Proteins and genes of different plant species involved in strigolactone biosynthesis pathway.

Protein	*Arabidopsis*	Rice	Pea	Petunia
*9-cis/*all-*trans*-P-carotene isomerase	*AtD27 {At1g03055)*	*D27 (Os11g0587000)*		
Carotenoid cleavage dioxygenase7	*MAX3 [At2g42620)*	*D17/HTD1 (Os04g0550600)*	*RMS5*	*DAD3*
Carotenoid cleavage dioxygenase8	*MAX4 {At4g32810)*	*D10 (0s01g0746400)*	*RMS1*	*DAD1*
Cytochrome P450, cytochrome711 (CYP711)	*MAX1 [At2g26170)*	*OsMAX1 (oslg0700900; Os01g0701500)*		*PhMAX1*

Strigolactones act as plant growth and development regulators affecting the plant architecture in response to various cues acting endogenously as phytohormones and exogenously in the rhizosphere. Given their dual role, SLs can be targeted for generating crops that are tolerant to various environmental stresses. In the following sections we will be discussing the SL biosynthesis, activity and functions including roles during plant development with special emphasis on plant stress responses and tolerance.

## Strigolactone Biosynthesis

### Lactone Containing Compounds

**FIGURE 1:**
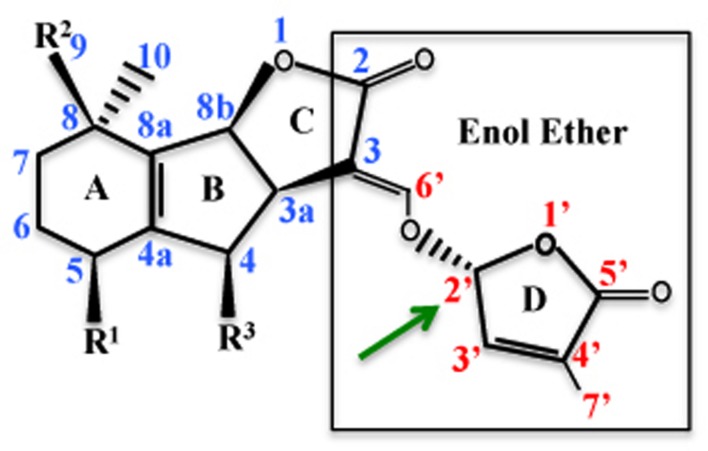
**Strigolactone (SL) structure: the structure of 5-DS is depicted showing the atom numbering.** 5-DS is composed of four ring A–D, where *R*^1^ is methyl group and *R*^2^ and *R*^3^ are hydrogen atoms. The C and D rings are connected with an enol ether bond and the D ring has a methyl group at C-4’ position. Structure activity relationship (SAR) studies have shown that C and D rings are important for SL activity. Stereochemistry at the C-2’ of the D-ring affects the bioactivity of SLs (Adopted from de Saint Germain et al., 2013).

With the advent of qualitative and quantitative techniques like liquid chromatography tandem mass spectrometry (LC-MS/MS), nearly 18 SLs have been identified and characterized on the basis of their structure. All the SLs are primarily composed of four rings (A–D), where the central tricyclic lactone (ABC rings) connects to a butenolide group (the D ring) via an enol ether bridge (**Figure 1**). SLs contain one to two methyl groups on the A ring and one or more hydroxyl or acetylonyl groups on the A- and B- rings. Due to the presence of variable side groups, the A and B rings show maximum divergence, whereas the C and D rings show maximum conservation (Xie et al., 2010). 5-deoxystrigol (5-DS) isolated from root exudates of *Lotus japonicus* L. is the simplest SL without any substitutions on the A and B rings. Owing to its simple structure and widespread distribution in both monocots and dicots, it is considered as the precursor for SLs, which are derived from 5-DS by hydroxylation, acetylation, oxidation, decarboxylation, ketolation, epoxidation, and dehydration reactions (Al-Babili and Bouwmeester, 2015).

Based on the presence and absence of tricyclic lactone ring (ABC ring), SLs are categorized into two types. Strigol-like and orobanchol-like compounds, contain the ABC ring. Whereas, β-ionone ring containing compound is known as carlactone (CL). All the 18 ABC-ring containing SLs contain a 2’*R*-configured butenolide ring (D-ring) and are distinguished by the differences in stereochemistry of B–C-ring junction (**Figure 1**). The C-ring in strigol-like SL derivatives of 5-DS is in β-orientation. Whereas orobanchol-like SLs are derived from *ent*-2-*epi*-5-DS with the C-ring in the α-orientation (Xie et al., 2010).

### Carotenoids

Carotenoids (40-Carbon) are isoprenoids, consisting of four terpene units, and are an integral component of photosynthetic membranes in all plants (Lu and Li, 2008). Catabolism of carotenoids results in the formation of apocarotenoids, which can both enzymatically and non-enzymatically cleave at the C-C double bonds into various carbonyl-containing metabolites such as retinoids, fungal pheromones, and ABA (Alder et al., 2008). Chemically all the SLs are sesquiterpene lactones and the structure is similar to terpenoids/isoprenoids, indicating that SLs are carotenoid derivatives. In plants, terpenoids/isoprenoids biosynthesis occurs primarily by the cytosolic mevalonic acid (MVA) pathway and the plastidic methylerythritol phosphate (MEP) pathway, where the MEP pathway leads to biosynthesis of monoterpenes, diterpenes, carotenoids, GAs, and ABA etc. In order to identify the SL biosynthetic pathway components, root exudates from both WT plants, treated with carotenoid biosynthetic pathway inhibitor, fluridone and maize carotenoid biosynthetic mutants such as *lemon white1* (*lw1*), *albescent plant1* (*al1y3*), *viviparous5* (*vp5*) were used for seed germination assays and quantitating SL content using LC-MS/MS. The root exudates from both treated WT plants and maize mutants showed low (10^-7^ to 10^-15^ M) SL content and a slower seed germination rate was observed. These results support the hypothesis that SLs are derived from carotenoids, probably using MEP pathway and may function as germination stimulants (Matusova et al., 2005).

Carotenoids were further established as the precursors for SLs by studying the enzymes required for catabolism of carotenoids, which include carotenoid cleavage dioxygenase (CCDs). CCDs catalyze oxidative cleavage of the double bond in 9-*cis*-epoxycarotenoids resulting in the formation of ABA. The first CCD to be identified was 9-*cis*-epoxycarotenoid dioxygenase (NCED), by analyzing the maize mutant *viviparous14* (*vp14*). Mutant analysis in various plant species revealed that CCD’s are also involved in SL biosynthesis in plants (Schwartz et al., 2004).

### RMS/MAX/D Pathway

Characterization of high branching/tillering mutants from different plants such as *more axillary growth* (*max1*, *max2*, *max3*, and *max4*) in *Arabidopsis*, *ramosus* (*rms1* to *rms5*) in pea, *dwarf*/*high tillering dwarf* (*d*/*htd*) in rice, and *decreased apical dominance* (*dad*) in petunia led to the identification of various components of SL biosynthetic and signaling pathways. Grafting and physiological experiments excluded the role of auxin or cytokinin, suggesting that the molecule responsible for branching phenotype exists in both stem and root and moves acropetally in the xylem (Leyser, 2009). Later cloning and sequencing revealed that *RMS5/MAX3/D17* (*HTD1*) and *RMS1/MAX4/D10/DAD1* encode carotenoid cleavage dioxygenases (CCD7 and CCD8, respectively), and *MAX1* encodes a cytochrome P450, CYP711A1 (**Table 1**; Schwartz et al., 2004; Booker et al., 2005). Biochemical analyses showed that these mutants are deficient in SLs and exogenous application of a synthetic SL, GR24, rescued the high branching phenotype of *max-3* and *max-4* but not of *rms4*, *max2*, and *d3* mutant plants, supporting that the former two are components of SL biosynthetic pathway and latter are the components of SL signaling pathway. The role of CCD7 in SL biosynthesis is further supported by analysis of branching phenotype in transgenic tomato plants expressing *SlCCD7* antisense constructs. These plants show an increased branching phenotype and lower level of SLs in the root exudates (Vogel et al., 2010). Biochemical studies in *Arabidopsis* revealed that AtCCD7 cleaves all-*trans*-β-carotene (C-40) into all-*trans*-β-apo-10′-carotenal (C27), which is subsequently cleaved by AtCCD8 to β-*apo*-13-carotenone (C-18 ketone), a bioactive product affecting root hair (RH) growth (Schwartz et al., 2004). Co-expression and sequential expression of *AtCCD7* and *AtCCD8* in *Escherichia coli* ordered the sequence of activity of the two enzymes during SL biosynthesis. *In vitro* studies confirmed that CCD8 from different plant species forms β-*apo*-13-carotenone from all-*trans*-β-apo-10′-carotenal (Alder et al., 2008).

**FIGURE 2:**
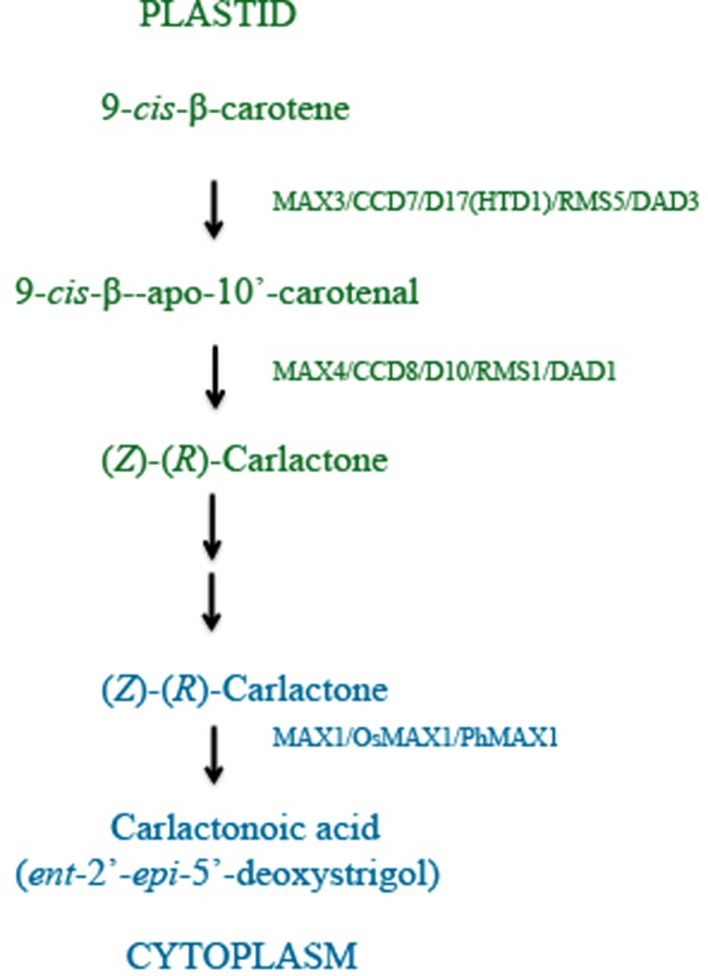
**RMS/MAX/D pathway: SLs are carotenoid derivatives and their biosynthesis takes place in plastids, where all-*trans*-β-carotene is enzymatically converted to carlactone (CL).** The enzymes which catalyze these steps have been identified and characterized from different plants and are listed in **Table 1** and include RMS (Pea), MAX (*Arabidopsis*), and D (Rice). CL is a mobile product, which is transported to cytoplasm and converted to carlactonoic acid (CLA) by action of MAX1. Subsequently CLA is converted to SLs with yet unidentified components of the RMS/MAX/D pathway.

Recent studies have identified *D27* as another component of SL biosynthesis pathway. Rice *OsD27* and its orthologs *AtD27* of *Arabidopsis* and *MtD27* of *Medicago truncatula* are implicated in SL biosynthesis based on the shoot branching phenotype observed in *d27* mutant plants, which is rescued by exogenous GR24 application (Lin et al., 2009; Waters et al., 2012a; Van Zeijl et al., 2015). Cloning and sequence analysis revealed that *D27* encodes a β-carotene isomerase localized in the plastids. This is supported by results from transient expression assays in onion epidermis cells and the presence of a functional plastid target sequence. Grafting experiments placed *D27* upstream of *MAX1*. The three enzymes, D27, CCD7, and CCD8 were ordered for the sequence in which they act during SL biosynthesis using *in vitro* biochemical experiments and found to function in the chloroplast (**Figure 2**). Moreover, a combination of purified D27, CCD7, and CCD8 proteins is found sufficient to convert all-*trans*-β-carotene into carlactone (CL). Exogenous CL is sufficient to rescue the high tillering and dwarf phenotype of rice mutants *d27* (*AtD27*), *ccd7* (*MAX3*), and *ccd8* (*MAX4*) suggesting that CL is the end product of steps catalyzed in the plastid (Alder et al., 2012).

Further evidence in support of CL as the product of CCD8 catalysis and as a precursor of SLs comes from structural analysis. CL contains A and D rings and the enol ether bridge suggesting that additional steps are required to add the B and C rings and conversion of CL to 5-DS and *ent*-2’-*epi*-5-DS (4-deoxyorobanchol, 4DO). It has been shown that *MAX1* catalyzes conversion of CL to SLs in the cytoplasm and *max1* mutant plants exhibit CL accumulation. It has been also shown that CL has biological activity similar to SLs including seed germination, acting as stimulant in root parasites, and regulator of shoot branching in higher plants (Seto et al., 2014).

To confirm MAX1 function, recombinant MAX1 was expressed in yeast microsomes and incubated with CL. It was found that MAX1 catalyzed the oxidation of CL to produce 9-desmethyl-9 carboxy-C2 or carlactonoic acid (CLA), confirmed by the detection of CLA and MeCLA (methyl ester carlactonoate) using LC-MS/MS. *In vivo* rescue experiments confirmed that both MeCLA and CLA are products of MAX1 as both can rescue the *max1* mutant phenotype. Interestingly, MeCLA and not CLA interacted with putative SL receptor, *DWARF14* (*AtD14*), supporting their role in SL pathway (Abe et al., 2014). Additionally, labeling experiments showed that MAX1 catalyzes the conversion of ^13^C-CL to ^13^C-2’-*epi*-5-DS and ^13^C-orobanchol (two main precursors of all the SLs), involving oxidation and dehydrogenation (Zhang et al., 2014).

**FIGURE 3:**
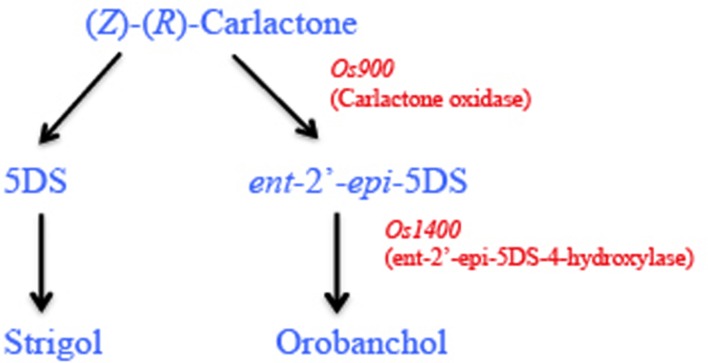
**Strigolactone biosynthesis in rice: rice (*Oryza sativa*) has five *MAX1* homologs (*Os900*, *Os1400*, *Os1500*, *Os1900*, and *Os5100*).** Carlactone (CL) the end product of CCD8 enzymatic activity in plastids is converted to orobanchol and strigol in the cytoplasm by the activity of MAX1. In rice, Os900 encodes a carlactone oxidase and catalyzes the conversion of CL to *ent*-2’-*epi*-5DS, which is subsequently converted to orobanchol by hydroxylation, catalyzed by Os1400.

Interestingly, the rice genome encodes for five *MAX1* homologs including *Os900* (*Os01g0700900*), *Os1400* (*Os01g0701400*), *Os1500* (*Os01g0701500*), *Os1900* (*Os02g0221900*), and *Os5100* (*Os06g0565100*) catalyzing two distinct steps in SL biosynthesis (Cardoso et al., 2014). Two of the homologs, Os900 and Os1400, present in the high tillering rice varieties that are low SL producers, catalyze two sequential steps during SL biosynthesis. Os900 catalyzes the oxidation of (*Z*)-(*R*)-CL to form, *ent*-2’-*epi*-5DS and Os1400 catalyzes the hydroxylation of *ent*-2’-*epi*-5DS to form orobanchol (**Figure 3**; Cardoso et al., 2014). Moreover, reconstitution experiments in *Nicotiana benthamiana* showed that *Os1400*, *Os900*, and *Os5100* produce small amounts of the precursor of orobanchol, consistent with the earlier findings where all the above three *MAX1* homologs repress shoot branching in *Arabidopsis max1* mutant plants. More recently, it has been demonstrated that transient expression of the four enzymes (Os900, Os1400, Os1500, Os5100) in *N. benthamiana* catalyzes the complete pathway from β-carotene to 5-DS (Zhang et al., 2014).

Identification of MAX1 homologs in woody perennial plant species *Populus tricocarpa* indicates the conservation of SL biosynthetic pathway across plant species (Czarnecki et al., 2014). A list of proteins encoded by genes involved in SL biosynthesis and SL perception is given in **Tables 1** and **2**, respectively.

**Table 2:** Genes encoding various proteins in different plant species involved in strigolactone perception/response pathway.

Protein	*Arabidopsis*	Rice	Pea	Petunia
α/β-Hydrolase	*AtD14*	*D14*/*D88*/*HTD2*		*DAD2*
F-box	*MAX2*	*D3*	*RMS4*	*PhMAX2APhMAX2B*
ClassI ClpATPase		*D53*		

### Expression of SL Biosynthetic Genes

Various analytical tools have been used to determine that roots have relatively high level of SLs as compared to other plant tissues such as hypocotyl, stem, and leaves (Cheng et al., 2013). In roots, the SL expression level was determined by transcript analysis of biosynthetic genes and it was found that rice *OsCCD7* (*HTD1*) and *OsCCD8* (*D10*) express in the vascular parenchyma cells. Similarly, in *Arabidopsis MAX1* is primarily expressed in the root vasculature and *AtCCD8* (*MAX4*) is expressed in the columella root cap of both primary and lateral roots (LRs). At*MAX2* and *OsD14* show high level of expression in the root elongation zone (Cheng et al., 2013). Recently, tomato *SlCCD7* was shown to be expressed at high levels in immature green fruits suggesting that SLs may have an additional function during fruit ripening and seed development (Vogel et al., 2010). Expression of SL biosynthetic genes in the roots corroborates with its role as a germination stimulant. Moreover, lower SL levels in shoots promote shoot branching.

### Structure Activity Relationship (SAR)

Structure activity relationship (SAR) studies have primarily helped to identify the bioactiphore of SLs. In the future such information can be useful in synthesizing various activity variants of SLs for studying their role during plant signaling mechanisms. Structurally SLs are composed of four rings (A–D; **Figure 1**), of which A–B rings are highly variable due to the presence of various side groups (*R*^1^ and *R*^2^). C and D rings are lactone heterocycles with a methyl at C-4’ position. SAR studies have highlighted the importance of the C and D rings, the hydroxyl group, and the stereochemistry in various SLs mediated responses. SAR studies have emphasized the requirement of C and D rings and methyl group on the D-ring for seed germination activity, supported by germination assays done with a variant of GR24, with the C and D rings hydrolyzed (Zwanenberg and Pospísil, 2013). Whereas GR25, a synthetic SL lacking the AB rings, suppresses shoot branching, supporting that the C and D rings is the bioactiphore contributing to the branch regulation activity of SLs (Umehara et al., 2015). Moreover, C and D rings are also required for hyphal branching in AMF symbiosis. Replacement of enol ether bond between C and D rings with an imino ether bond shows little decrease in activity, suggesting that the kind of linkage is not important for SLs activity (Akiyama et al., 2010). D-ring modification reduces SL bioactivity during seed germination in root parasites, suggesting that the bioactiphore of SLs for induction of seed germination also lies in the C and D-rings part of the molecule (Zwanenberg and Pospísil, 2013).

All SLs are categorized into two groups of diastereoisomers based on the stereochemistry at C-2’, namely strigol-type and orobanchol-type SLs (**Figure 1**). The (2)-CL can exist stereochemically as either *R* or *S*. In rice and *Arabidopsis* only the *R* configured (*Z*)-CL has been detected, a precursor for all SLs *in vivo* (Seto et al., 2014). C-2’-(*R*) stereochemistry also affects the germination activity of SLs. The stereoisomers whose configuration at position C-3a, C-8b, and C-2’ are (*R*), (*S*), and (*R*), respectively, are most active SLs (**Figure 1**; Sugimoto et al., 1998). Though there are some exceptions, for example 2’-epiorobanchol is slightly more active than orobanchol (Xie et al., 2007). Recently it has been shown that the configuration at C-2’, determining the steric position of the D-ring relative to the enol ether olefin bond, is critical for the bioactivity in rice. Substitution of an enol ether moiety by an alkoxy or imino ether results in reduced biological activity in rice. Moreover, yeast two-hybrid (Y2H) assays confirmed that the 2’*R* configuration is necessary for the interaction with DWARF14 (D14), a putative SL receptor and DWARF53 (D53), a repressor of SL signaling pathway in rice (Jiang et al., 2013).

Heterogeneous results have been obtained as far as the configuration at C-2’ and its importance in SL activity during shoot branching is concerned. Stereochemistry at C-2’ is not an important structural feature for pea shoot branching. Moreover, in pea both the D-ring and α/β-unsaturation at C-C bonds are essential for SL biological activity (Boyer et al., 2012). Presence of an intact D-ring with an enol ether bridge with C-ring is essential for the inhibition of tiller-bud outgrowth and the (*R*) configuration at C-2’ also influences SL activity in rice and *Arabidopsis* (Umehara et al., 2015). The structural requirement for branch inhibition lies in the C and D rings, any minor modification in these rings such as replacement of C-C double bond at C3’C6’ or C3’C4’ and substitution at C2’ leads to reduction in bioactivity. For branch inhibition, stereochemistry at C2’ has no effect on bioactivity but affects hyphal branching in AMF (Boyer et al., 2012). SAR studies have established the importance of the C and D rings, 2’*R* configuration, and the enol ether bridge in various SL regulated biological activities.

## Regulation of Strigolactone Biosynthesis

A comprehensive knowledge of the regulatory mechanisms in SL biosynthesis provides tools to manipulate both the SL biosynthetic and response pathway genes for the benefit of the plant growth. Previous studies have shown that SL biosynthesis is regulated both at post-transcriptional and post-translational levels by various exogenous and endogenous factors described in this section.

### Plant Hormones Mediated Regulation

Auxins positively regulate expression of CCD7 and/or CCD8 in pea, *Arabidopsis*, rice, and Chrysanthemum (*Dendranthema grandiflorum*). In pea, auxins positively regulate the *RMS5* (*CCD7*) and *RMS1* (*CCD8*) transcript levels as supported by experiments including exogenous application of auxins, decapitation of the apical shoot tip, and using auxin transport inhibitors. Similar results were obtained in *Arabidopsis*, where auxins maintain *AtCCD7*/*MAX3* and *AtCCD8*/*MAX4* transcript levels and probably SL levels (Brewer et al., 2009; Hayward et al., 2009; Liang et al., 2010). Mutant studies provide insight into the mechanism of auxin mediated transcriptional regulation. *AtCCD7* and *AtCCD8* transcript levels are reduced in *axr1* and *bdl* mutants, where *AUXIN RESISTANT1*(*AXR1*) encodes a protein highly similar to ubiquitin-activating enzyme E1 and *BODENLOS* (*BDL*) encodes *INDOLEACETIC ACID RESPONSE12* (*IAA12*) a transcription repressor. AXR1 protein is required for the stabilization of the SKP1-CUL1 E3 ubiquitin ligase complex or SCF^TIR1/AFB^, composed of TRANSPORT INHIBITOR RESPONSE1 (TIR1) an F-box protein and Auxin-related F-box (AFB), an auxin receptor. Mutation in *AXR1* causes changes in this complex and the downstream events regulated by auxins, which might include SL biosynthesis. SCF^TIR1/AFB^ complex primarily mediates degradation of BDL protein. These results are further partially supported by phenotypic analysis of various combination mutants of the auxin signaling pathway and the SL signaling pathway. Exogenous application of SLs suppresses the branching phenotype in *Arabidopsis axr1*, quadruple (*tir1afb1,2,3*), and *bdl* mutant plants. Moreover, promoter analysis revealed the presence of auxin responsive elements in both *CCD7* and *CCD8* genes, further supporting auxin mediated regulation of SL biosynthesis (Hayward et al., 2009). Studies in *axr1* mutant plants showed that expression levels of *MAX3*, *MAX4*, and *D27* are reduced in response to auxin depletion, achieved via decapitation and NPA (Naphthylphthalamic acid) treatment, where NPA inhibits auxin transport. Moreover, *MAX3* and *MAX4* transcript levels are upregulated in *d27* mutants, suggesting feedback regulation by SLs itself. Exogenous application of NAA results in upregulation of *CCD8* expression in the (pro) vasculature tissue of the primary roots (PRs) and cortical tissue of the root apex elongation zone and decapitation leads to decreased expression of *CCD7* and *CCD8* (Rasmussen et al., 2012). Auxin signaling using the AXR1 pathway up-regulates the expression of SL biosynthetic genes simultaneously down-regulating CK biosynthesis. Both these hormones regulate bud outgrowth by regulating expression of *TEOSINTE BRANCHED-CYCLOIDEA-PCP* (*TCP*) family transcription factor (TF) *BRANCHED1* (*BRC1*) that are known to be required for inhibiting branching. However, for pea and *Arabidopsis brc1* mutants, the high branching phenotype cannot be rescued by SL treatment, indicating that *BRC1* acts downstream of the SL biosynthetic pathway (Brewer et al., 2009; Braun et al., 2012; Dun et al., 2012). Taken together these results indicate a positive regulatory role of auxins on SL biosynthesis.

A correlation is also found between ABA and SL biosynthesis as both the hormones are derived from carotenoids. Studies done in WT tomato plants treated with AbaminSG, an inhibitor of NCED (an ABA biosynthesis enzyme) and ABA mutants including *notabilis* (mutated in NCED) and *sitiens* and *flacca* (mutated in AAO, Aldehyde oxidase) showed reduced levels of SLs, assessed by LC-MS/MS. Moreover, reduction in SL production correlated with down-regulation of *LeCCD7* and *LeCCD8* genes in the three tomato mutants (López-Ráez et al., 2010). Additional evidence in support of ABA as a SL biosynthesis regulator was based on the lower germination rates of *Striga hermonthica* and *Phelipanche ramosa* seeds in the presence of ABA-deficient mutants of maize (*viviparous 14*, *vp14*) and tomato (*notabilis*). LC-MS/MS analysis of root exudates of tomato confirmed a reduced SL level in these mutants, though it is not clear whether NCED or ABA is responsible for reduced SL levels (López-Ráez et al., 2010). These studies support the hypothesis that ABA positively regulates SL levels.

### AMF and Nodulation Mediated Regulation

In tomato, SL levels increase in response to AMF, which correlates with an induction of *SlCCD7* (Vogel et al., 2010). Nodulation is also promoted in response to legume and AMF symbiosis, probably involving SLs. It has been found that *NODULATION SIGNALING PATHWAY 1* (*NSP1*) and *NSP2*, GRAS-type TFs, are essential for nodule formation in rhizobium during symbiosis (Liu et al., 2011). GRAS-type TFs, named after the first three members: *GIBBERELLIC-ACID INSENSITIVE* (*GAI*), *REPRESSOR OF GAI* (*RGA*), and *ECROW* (*SCR*) are known for their regulatory role in root and shoot development, GA signaling, phytochrome A signal transduction, and nodule morphogenesis. Moreover, *NSP1* and *NSP2* are also indispensable for SL biosynthesis under non-symbiotic conditions in *M. truncatula* and rice. Double knockout mutants in *M. truncatula* (*nsp1nsp2*) and *Oryza sativa* (*Osnsp1Osnsp2*) do not produce detectable levels of SLs. Moreover, in these lines a highly reduced expression of *MtD27* and *OsD27* is observed. Interestingly, for regulating SL biosynthesis *NSP1* and *NSP2* do not use the calcium calmodulin kinase pathway, which is canonically required for nodulation in legumes. These plants also show reduced AMF symbiosis, suggesting that *NSP1* and *NSP2* are also activated in response to AMF to regulate SL biosynthesis (Liu et al., 2011).

### miRNA Mediated Regulation

miRNAs are 21–24 nucleotide long sequences complementary to the target mRNA, which upon binding to target mRNA leads to its degradation, and subsequently repression of translation. In *Arabidopsis* (*MAX1*, *MAX3*, *MAX4*) and rice (*D27*, *D3*, *D10*), all the SL pathway genes possess target sequences recognized by *miR156a-g*. *miR156* regulates plant architecture and development, which are influenced by the environment. Overexpression of *miR156* in *Arabidopsis*, rice, and maize causes increased shoot branching phenotype, a hallmark of SL biosynthesis and response mutants. Reverse Transcription-Polymerase chain reaction (RT-PCR) in *osa-miR156* overexpressing rice plants show reduced *D27* transcript levels, suggesting *osa-miR156* negatively regulates *D27*. Interestingly, these plants also exhibit elevated levels of *D3* and *D14* transcripts, pointing toward feedback regulation of the SL pathway genes (Chen et al., 2015). Taken together these results suggest the existence of a new pathway regulated by *miR156* not requiring *O. sativa SQUAMOSA-PROMOTER BINDING PROTEIN-LIKE* (*OsSPL*) in controlling apical dominance and tillering outgrowth. *SPL* proteins in maize and rice are known to regulate morphological development (Luo et al., 2012).

### Regulation under Nutrient Deficiency

Inorganic nutrients such as phosphates and nitrates are major sources of Nitrogen (N) and Phosphate (P) and are known to affect plant growth and architecture, which depends on root and shoot growth (Linkohr et al., 2002). There is ample evidence supporting the role of plant hormones such as auxin and CKs in nutrient signaling pathways leading to modulation of plant architecture (Leyser, 2009). Research in the last decade has implicated SLs in nutrient signaling pathways. Extremely low level of SLs is detected in plants growing under standard laboratory conditions, whereas sub-optimal conditions like insufficient nutrients, enhances SL levels, probably endowing plasticity to plants to adapt to these conditions (Kohlen et al., 2011). In LC-MS/MS analysis, an elevated level of SLs in root and root exudates under phosphate and nitrate deficient conditions is detected. For example, leguminous plants show increase in SL levels in response to both Pi and N -deficient conditions (Foo et al., 2013; Sun et al., 2014). Collectively, all these observations suggest that SLs may function as second messenger molecules during nutrient deprivation conditions. Moreover, biosynthesis of SLs is regulated by auxins (Hayward et al., 2009). A cross regulation between auxin and SLs is also supported by RNA-seq analyses of maize root transition zone (TZ), where under *N*-deficient conditions, both SL biosynthetic genes and transportation genes are down-regulated along with Adaptor Protein-2 (AP-2). AP-2 is required for clathrin- mediated endocytosis, which is involved in auxin signaling and PINFORMED1 (PIN1) mediated transport. SLs have been shown to regulate auxin transport via AP-2 and PIN1 protein (Trevisan et al., 2015). These results indicate that down-regulation of SLs in the TZ could be the early response to nutrient insufficient conditions.

Under Pi-deficient conditions, WT *Arabidopsis* plants show inhibition of lateral bud outgrowth and increase in orobanchol levels in the root and xylem sap, respectively. Because *Arabidopsis* is a non-host for AMF, therefore SLs probably primarily increase the efficiency of Pi usage by modulating plant architecture (Kohlen et al., 2011; Mayzlish-Gati et al., 2012). Similar results were obtained in rice under P-deficient conditions, where 2’-*epi*-5-DS levels are increased and tiller bud outgrowth is inhibited. Such an effect is not observed in *d3* and *d10* rice mutants, suggesting that SL biosynthesis and not the perception pathways are required during Pi-deficient conditions (Umehara et al., 2010). SLs not only affect the shoot architecture but also root architecture in response to Pi availability. Pi deficient conditions in *Arabidopsis* result in shorter PR length, repressed LR formation, and reduced meristem cell number in both SL biosynthetic and SL signaling mutants as compared to WT plants. The Pi deficiency phenotypes are rescued by GR24 in all genotypes except the SL signaling mutants, suggesting that SL biosynthesis is required under these conditions. SL mediated regulation of root architecture requires auxins by controlling the localization of PIN proteins (Shinohara et al., 2013). Moreover, under Pi-deficiency, *max2* and *max4* show altered expression of Pi-deficiency hallmark genes including type 5 acid phosphatase (*ACP5*), phosphate transporter 1;5 (*PHT1;5*), and *PHT1;4.* Auxin transporter gene, *TIR1* was shown to be involved in the SL-mediated response to low Pi, suggesting SLs and auxins coordinate the response to low Pi (Mayzlish-Gati et al., 2012). In rice *Os900* is upregulated under Pi-deficiency, whereas *Os1400* expression remains unchanged under the same condition (Cardoso et al., 2014). Recent work in *O. sativa* has shown that SL biosynthesis is up-regulated during N and Pi –deficient conditions. In WT plants, low N and Pi causes increased root length and decreased LR density. Whereas, in the SL biosynthetic and response mutants including *d10*, *d27*, and *d3* roots are insensitive under deficient conditions. Expression analysis revealed up-regulation of *D10*, *D27*, and *D3* under N and Pi-deficient conditions. Moreover, exogenous application of GR24 restored the insensitive phenotype in all the mutants except *d3* suggesting that SLs biosynthesis is induced under Pi and N -deficiency. Auxins are also implicated in SL-mediated response as transport of radiolabelled IAA (indole-3-acetic acid) and activity of *DR5::GUS5*, an auxin reporter is reduced in GR24 treated WT, *d10*, and *d27* plants (Sun et al., 2014).

### Light Induced Expression

A connection between light and SLs comes from the studies performed in various plants (reviewed by Koltai and Kapulnik, 2011). Studies in *Arabidopsis* revealed that light signaling related genes are induced after exposure to GR24. Whereas, in the SL-deficient mutants, light regulated genes were down-regulated (Mashiguchi et al., 2009). Similar results were obtained using WT and SL-deficient *SlORT1* tomato plants, which are deficient in SL production and have reduced chlorophyll levels and light harvesting complex (LHC) genes. Treatment with GR24 causes increased chlorophyll levels and increased expression of LHC genes (Mayzlish-Gati et al., 2010). Moreover, increasing light intensity positively regulates *SlCCD7* expression level (Koltai et al., 2011). SLs have been shown to regulate nuclear localization of Ubiquitin ligase (COP1), which partially controls Elongated Hypocotyl 5 (HY5), a light regulator protein, mimicking light-adapted *Arabidopsis* seedling growth (Tsuchiya et al., 2010; Koltai et al., 2011).

A correlation between light and SLs is evident from the phenotypic analysis of *Arabidopsis* plants grown under low light intensity and crowded conditions, exhibiting elongated leaves with long and slender stems. Whereas, SL mutant plants such as *max2*, *max1* of *Arabidopsis*, and *rms3* and *rms4* of pea have round leaves and are of short stature (Stirnberg et al., 2002). Moreover, *Arabidopsis phytochromeB* (*phyB*) mutant plants have poor response to the high red light condition resulting in tall slender plants with reduced branching. A role for SLs in the phytochrome pathway was evident by double mutant analysis using *phyB* and *max2*, *max4*, or *brc1* under high red light conditions, where the *phyB* phenotype was repressed. The phenotype observed is similar to *max2*, *max4*, and *brc1* single mutants of high branching, indicating that SLs acts downstream of *PHYB* in the high red light response (Finlayson et al., 2010). Conversely, under low red light the *phyB* phenotype repression is relieved. This hypothesis is not fully supported by *brc1* mutant analysis, which exhibits a higher number of branches under low red light conditions. Interestingly, auxin production is increased in shaded plants (Tao et al., 2008). Based on these observations one might speculate that increased auxin production leads to increased SL biosynthesis, promoting a shade avoidance phenotype. A correlation between light and SLs is demonstrated in SL-deficient pea mutants, *Psccd8* and *Psccd7*, producing significantly fewer adventitious roots (ARs) than WT seedlings when grown in the dark, but not when grown in the light. However, in *Arabidopsis* seedlings, SL-insensitive *max2* mutants had altered light-induced seed germination and photomorphogenesis (Tsuchiya et al., 2010).

## Strigolactones and Plant Growth and Development

### Rhizosphere

Strigolactones secreted in the root exudates act as germination stimulants in parasitic weeds. Parasitic weeds attach to the root of host plant by means of a specialized structure called the haustorium, which retrieves nutrients and water from the xylem tissue causing extensive yield loss in crop plants globally. Initially, SLs were identified as lactone containing compounds such as strigol, strigyl acetate, and sorgolactone acting as germination stimulants for *Striga* spp. (witchweeds; Orobanchaceae) secreted in the root exudates of tropical cereal crops such as *Gossypium hirsutum* (cotton), *Vigna unguiculata* (cowpea), *Zea mays* (maize), *Pennisetum glaucum* (pearl millet), *Sorghum bicolor* (sorghum), and *O. sativa* (rice) (Cook et al., 1972; Hauck et al., 1992; Siame et al., 1993). Later, alectrol and orobanchol were identified as germination stimulants for *Orobanche* spp. (broomrapes; Orobanchaceae), parasitizing the temperate climate crops including *Lycopersicon esculentum* (tomato), *Helianthus annuus* (sunflower), *Solanum tuberosum* (potato), *Brassica napus* (rapeseed), and *N. tabacum* (tobacco) (Yokota et al., 1998).

A more beneficial role of SLs for plants was discovered in hyphal branching in AMF of glomeromycota. AMF are obligate biotrophs, colonizing plant roots to establish symbiosis. AMF use specialized structures called arbuscules to receive photosynthates from plant and in turn provide water, phosphate, and nitrogen to the plant. The root exudates from plants are able to induce hyphal branching in AMF and initially these compounds were called branching factors (BFs). Later, the BFs were characterized as SLs, and their functional characterization using a synthetic analog, GR24, showed induction of hyphal branching in AMF, *Gigaspora margarita*, confirming the role of SLs in hyphal branching (Akiyama and Hayashi, 2006).

Root nodulation is a symbiotic relation between leguminous plants (Fabaceae family) and nitrogen-fixing bacteria (*Rhizobium*) for fixing atmospheric nitrogen (N) under N-deficient condition. Studies in pea have implicated SLs in maintaining the nodule number. Determination of SL levels and nodule number in SL-deficient *rms1* pea plants revealed that the root exudates and tissue are almost completely deficient in SLs and have 40% fewer nodules than WT plants. Moreover, GR24 treatment rescues the nodulation defect, restoring the WT nodule number. Grafting studies revealed that nodule number and SL levels in root tissue of *rms1* are unaffected by grafting to WT scions indicating that SLs in the root regulates nodule number, ruling out the role for shoot derived SLs (Foo and Davies, 2011). Similar studies in *M. truncatula* showed that SLs regulate nodule number in dosage dependent manner. Lower concentration (0.1 μM) of SLs increased and higher concentration (2–5 μM) decreased nodule number. Moreover, expression of nodulation marker, *NOD1* (*EARLY NODULATION11*) is down-regulated in GR24 treated plants (De Cuyper et al., 2015). Based on these results, SLs were proposed to act as regulators of nodulation in leguminous plants.

### Shoot Branching

Strigolactones function in shoot branching was unveiled by isolation and characterization of increased shoot branching mutants from various plant species such as *decreased apical dominance1* (*dad1*) in *Petunia hybrida*, *ramosus1* (*rms1*) to *rms5* in *P. sativum*, *more axillary growth1-4* (*max1-4*) in *Arabidopsis*, *dwarf* and *high tillering dwarf* (*d*/*htd*) in rice (Leyser, 2009; Beveridge and Kyozuka, 2010). Quantification of hormone levels ruled out the involvement of auxin and CK in the branching phenotype. Subsequent cloning and sequencing revealed involvement of SL biosynthesis genes *RMS5*/*MAX3*/*D17* (*HTD1*) and *RMS1*/*MAX4*/*D10*/*DAD1* encoding carotenoid cleavage dioxygenases CCD7 and CCD8, respectively. Similarly, *MAX1*/*PhMAX* encoding a cytochrome P450, CYP711A1 and *RMS4*/*MAX2*/*D3* encoding an F-box protein, involved in SL biosynthesis and signal perception, respectively, were identified. The role of SLs in branching is further supported by studies done in pea and rice *ccd8* mutants, *rms1* and *d10*, respectively. Both mutants are deficient in SLs and the branching phenotype is rescued by treatment with GR24 and natural SLs. On the other hand, when GR24 is applied to signal perception mutant *rms3* of pea and *d3* of rice, the branching phenotype is not rescued (Gomez-Roldan et al., 2008; Umehara et al., 2015). Tomato plants expressing *SlCCD7* antisense constructs show excessive shoot branching phenotype and reduced levels of SLs. These plants showed higher expression levels of SL biosynthesis genes in unripe fruits, suggesting additional SL function in fruit ripening or seed development (Vogel et al., 2010). All these studies established SLs as a negative regulator of branching.

### Rooting

Primary root (PR) depends upon the activity of root apical meristem (RAM). It has been shown that the cell division, elongation, and differentiation process in RAM are regulated by SLs, CKs, and auxins. The PR length in SL mutants *max1*, *max2, max3*, and *max4* is shorter than in WT plants, a phenotype rescued by GR24 application in the SL biosynthetic mutants (*max1*, *max3*, and *max4*) but not in the SL perception mutant (*max2*). Studies have also shown that SLs repress LR formation and promote RH elongation (Kapulnik et al., 2011; Ruyter-Spira et al., 2011). SLs may affect LR formation via changes in auxin eﬄux by regulating PIN proteins where auxin distribution determines LR position, initiation, and elongation (De Smet, 2012; Koltai, 2014). Furthermore, genetically SLs are placed downstream of auxin based on studies done in auxin and SL signaling mutants (Mayzlish-Gati et al., 2012). SLs and CKs act as suppressors of AR formation, supported by the more AR formation phenotype of SL mutants in pea and *Arabidopsis*, with lower CK levels in xylem. Interestingly, auxins also play a pivotal role during AR development (Rasmussen et al., 2012). This suggests the existence of a crosstalk between SLs, auxins, and CKs to orchestrate AR development.

### Senescence

Senescence is influenced by various exogenous factors such as drought, high temperature, and biotic and abiotic stresses and endogenous factors primarily include plant hormones. Plant hormones like ABA, JA, and ET are known inducers of senescence, whereas CKs inhibit senescence (Jibran et al., 2013). During senescence, nutrients are reallocated from older tissue to the growing and younger tissue. Recently studies conducted in *Arabidopsis oresara9* (*ore9*)/*max2* and rice *d3* mutants, which exhibit delayed senescence, suggest a role for SLs during senescence. Similarly, transgenic *L. japonicus*, silenced for *LjCCD7*/*MAX3* show delayed leaf senescence and increased branching (Yan et al., 2007; Czarnecki et al., 2013; Yamada et al., 2014). In rice, GR24 restores normal leaf senescence in SL-deficient mutants (*d10*, *d27*, and *d17*), whereas it has no effect on SL response mutants (*d3* and *d14*). Moreover, GR24 positively regulate SAG (Senescence Associated Gene) expression in both WT and SL mutant plants. Additionally, it was found that SLs regulate leaf senescence in response to Pi-deficient conditions (Yamada et al., 2014). Taken together these results indicate to a role for SLs during senescence.

## Regulatory Mechanisms of Strigolactone Signaling

### Trafficking

Previously, it has been shown that SL signaling affects auxin flux in the root tip thereby affecting LR formation, PR meristem size, and RH elongation (Koltai, 2014). Auxin flux in turn depends on the localization of PIN protein, auxin transporter, in the plasma membrane (PM). PIN1 localization and trafficking is dependent on filamentous-actin (*F*-actin), as stabilization of *F*-actin slows PIN1 trafficking (Shinohara et al., 2013). Insight into the mechanism of action of SLs came from root elongation assays done in *Arabidopsis* mutant plants *max2*, *eir1* (PIN2), *der1* (actin), and *tir3* (*transport inhibitor response3*). GR24 treatment in WT plants show increased endocytosis supported by ARA-7 (plant Rab5 small GTPases) labeled vesicles and reduced *F*-actin bundling. Whereas, mutant plants show increased polar localization and accumulation of PIN2 in brefeldin A (BFA) bodies, where BFA is a vesicular transport inhibitor. Moreover, there is increased PIN2 transcription, endocytosis, and actin cytoskeleton reorganization. *max2* plants exhibit none of the above phenotypes upon treatment with GR24. *der1* and *tir3* plants display reduced sensitivity to GR24 with respect to RH elongation (Pandya-Kumar et al., 2014). Similar results were obtained by monitoring the levels of PIN1 at the PM in *Arabidopsis* shoot. SLs accelerate PIN1 removal from the PM causing the shoot branching phenotype. Additionally, PIN1 levels are also depleted in the PM of xylem parenchyma cells in the stem and this process is clathrin mediated (Shinohara et al., 2013). In conclusion, SLs act as positive regulator of PIN protein localization, transcription, translation, and trafficking by reorganizing actin cytoskeleton modulating auxin distribution. Auxin in turn, positively regulates SL biosynthesis (Hayward et al., 2009).

### Transcription

A crosstalk between SLs and plant hormones including ABA and CKs is evident by comparative transcriptome analysis of *max2* and WT *Arabidopsis* plants under well watered and dehydration conditions. *max2* plants exhibit down-regulation of *AtNAC2* (a NAC TF), which is inducible by *CIPK1* (*CBL*-*INTERACTING PROTEIN KINASE1*), ABA, ET, and auxin signaling (He et al., 2005). Moreover, *AtNAC2* is known to be involved in LR development, which is also a SL regulated process. *max2* plants also show reduced expression of *ABCG22* and *ABCG40*, ABA import genes. Previously, *abcg22* and *abcg40* plants were shown to be drought sensitive due to reduced stomata closure and increased transpiration, supporting the positive regulatory role of ABA in drought signaling responses (Osakabe et al., 2014). CKs are known to enhance drought tolerance and SLs have been shown to regulate expression of *CRX* genes, encoding CK oxidase/dehydrogenase, required for CK catabolism. The *CRX* genes (including *CKX1*, *CKX2*, *CKX3*, and *CKX5*) are down-regulated in *max2* plants (Reguera et al., 2013; Ha et al., 2014).

Biochemical approaches have suggested two classes of TFs as downstream targets of *MAX2*, including *bri1*-EMS-suppressor 1 (*BES1*; BA activated TF) and *DELLA* (GA activated TF), where the former interacts with MAX2 (Nakamura et al., 2013; Wang et al., 2013). Moreover, AtD14, a putative SL receptor, promotes BES1 degradation and *BES1* knockdown suppresses the branching phenotype of the *max2-1* mutant. Another TF, *BRC1* (*BRANCHED1*) of pea, is regulated by SLs. It is a homolog of *TB1* of maize and *AtBRC1* of *Arabidopsis* (Braun et al., 2012). *Psbrc1* mutants have increased the GR24-resistant branching phenotype, placing *BRC1* downstream of SLs. Whereas rice *FC1* (*FINECULM1*), a *BRC1* homolog, is not SLs responsive, double mutant analysis shows that SL signaling and *BRC1* effects on shoot branching are at least partially independent in rice, *Arabidopsis*, and pea (Minakuchi et al., 2010).

Recent studies in rice have identified DWARF53 (D53), a class I Clp ATPase (Jiang et al., 2013). D53’s structural analysis has revealed the presence of three Ethylene-responsive element binding factor-associated Amphiphilic Repression (EAR) motifs. EAR motifs have been shown to be involved in transcriptional repression to regulate plant gene expression. EAR motifs are found in proteins that interact with topless related (TPR) transcription co-repressor (Kagale and Rozwadowski, 2011). D53 has been proposed to regulate SL-responsive genes in similar manner. However, evidence in support of this mode of D53 mediated regulation is insufficient because D53 binds weakly to TPR proteins (Zhou et al., 2013). Besides, EAR motifs have been also implicated in interaction with CTLH-domain containing protein. In *Arabidopsis*, CTLH-domain containing proteins are implicated in cytoskeleton reorganization and endocytosis (Kagale and Rozwadowski, 2011). SLs have been shown to regulate auxin localization and transport via trafficking and cytoskeleton rearrangements (Pandya-Kumar et al., 2014).

### Proteolysis

Proteolysis is a post-translational regulatory mechanism involving RING-finger E3 ligases including Anaphase promoting complex (APC), and Skp1-Culin-F-box protein (SCF) complex to regulate normal cellular homeostasis. One of the regulatory mechanism of SL signaling requires the Leucine-rich repeat F-box protein, ORE9/MAX2/RMS4/D3, which acts as a substrate recruiting subunit of SCF-type ubiquitin E3 ligase and an α/β-fold hydrolase D14/D88/HTD2 of rice and DAD2 of Petunia, which might act as the probable SL receptor (Chevalier et al., 2014). Both *max2* and *d14* mutants exhibit a highly branched phenotype and are SL insensitive, supporting their function in SL perception pathway (Waters et al., 2012b). These two proteins act analogous to GA signaling, where GA binds to its receptor, GIBBERELLIN INSENSITIVE DWARF1 (GID1), promoting formation of the GA-GID1-DELLA complex, where DELLA protein is a TF. The GA-GID1-DELLA complex is recognized by the SCF^SLY 1/GID2^ complex, composed of SLEEPY1 (SLY1), a F-box protein, recruiting DELLA proteins for proteasomal degradation (Gallego-Bartolomè et al., 2012). The role of MAX2 in the SCF complex is further supported by studies in *Arabidopsis*, where *ORE9*/*MAX2* has been shown to possess a functional F-box domain. *CaMV35S::MAX2* (deleted in F-box domain) construct is unable to complement the *max2* mutant phenotype. Moreover, Myc tagged MAX2 interacts with core SCF subunits Skp1-like *Arabidopsis* protein (ASK1) and *Arabidopsis* Culin (AtCUL1) (Stirnberg et al., 2007). Structural analysis revealed similarities between D14/DAD2 proteins with the GA receptor GID1. GID1 and D14 are members of the α/β-hydrolase family and SLY1/GID2 and MAX2/D3 are members of the F-box family. Therefore, D14 and MAX2/D3 can function in SL signaling in a manner similar to that of GID1 and SLY1/GID2 in GA signaling. Interestingly, SLENDER RICE1 (SLR1), a rice DELLA protein, has been proposed as potential target of the SL signaling (Zheng et al., 2014).

Analogous to GID1 function, GR24’s binding and hydrolysis by DAD2 (D14) of petunia produces two compounds: ABC-OH and D-OH. Moreover, the hydrolytic activity of DAD2 is a prerequisite for the protein–protein interaction of the DAD2-GR24 complex with PhMAX2 (Petunia ortholog of MAX2). The crystal structure of DAD2 confirmed the presence of a catalytic cavity in which SLs can fit and mutant analysis confirmed three highly conserved amino acid residues required for DAD2/D14 activity (Hamiaux et al., 2012). D14-mediated hydrolysis of SLs results in activation of downstream targets such as SLR1, a rice DELLA protein. DELLA proteins have been shown to negatively regulate GA signaling by interacting with GID1. Addition of D-OH alone does not result in D14-SLR1 complex formation in Y2H assay and neither inhibited the high tillering phenotype in *d27-1* mutant supporting the hypothesis that hydrolysis and binding of SL with D14 is a pre-requisite for SL signaling (Nakamura et al., 2013). Given the similarities between GA and SL signaling pathways, a crosstalk could be speculated and this is supported by overexpression of *GA2-OXIDASE* gene in rice, which reduces GA levels and enhances tillering, a phenotype similar to SL deficient mutant plants (Lo et al., 2008).

Rice D53 is a Clp ATPase, a family of proteins known for their function in protein degradation and disaggregation (Jiang et al., 2013). D53 is a homolog of *Arabidopsis* SUPPRESSOR OF MORE AXILLARY GROWTH2 (SMXL) family protein, a target of MAX2-D14-dependent protein degradation. A specific five amino acid motif deletion in D53 results in dominant a SL-resistant increased branching phenotype, whereas knockdown of *D53* suppresses the *d3* (rice ortholog of *MAX2*) and *d14* mutant phenotypes indicating that *D53* acts as a negative regulator of SL signaling. Besides, an interaction has been confirmed between D53, D14, and D3 by Y2H (Hamiaux et al., 2012). Moreover, D53 is rapidly degraded in the presence of GR24 (Zhou et al., 2013). The data above suggests that D53 is a target of SL signaling in shoot branching and acts as a negative regulator of the SL response. This seems to be a canonical SL signaling mechanism, as a similar pathway exists in karrikin signaling, where karrikin acts through KAI2, a close relative of D14 and MAX2 and is negatively regulated by SMAX1 (Smith and Li, 2014).

### Localization and Transport

Early grafting experiments showed that SLs are transported from roots to shoot in the xylem of *Arabidopsis* and tomato, which provided insight into SL signaling regulation via localization and transport (Kohlen et al., 2011). Roots serve as the primary site for SL biosynthesis, from where SLs are either exuded out into the rhizosphere or transported via xylem to different plant parts. Recent work has implicated *Petunia hybrida PLEIOTROPIC DRUG RESISTENCE I* (*PDR1*), an ATP-binding cassette subtype G (ABCG)/pleiotropic drug resistance (PDR) type transporter, functioning in eﬄux of SLs into the rhizosphere. Interestingly, in *pdr1* mutant plants, SL levels in root extract is similar to WT but severely reduced in root exudates, suggesting *PhPDR1* acts in SL exudation and probably there is feedback regulation to maintain SL levels. Phenotypically enhanced lateral bud outgrowth and reduced interaction with AMF is observed in *pdr1* plants, indicating that *PDR1* mediates SL transport within the plant as well. Moreover, *PhPDR1* expression in roots is induced by P-deficiency, AMF colonization, and treatment with GR24 and NAA. Localization of *PaPDR1* in the PM of the sub-epidermal cells of the LRs in *Arabidopsis* further supported their role in eﬄux of SLs in the rhizosphere (Kretzschmar et al., 2012). It was shown that when *PaPDR1* is co-expressed with *DAD1* (*CCD8*) it localizes to the apical membrane of root hypodermal cells (HPCs) and might mediate acropetal transport. In the hypodermal passage cells, an entry point for mycorrhizal fungi, *PaPDR1* is present in the lateral membranes, probably transporting SLs to the soil. Moreover, a *papdr1* mutant is impaired in SL transport both to the shoot-tip and rhizopshere. The functionality of *GFP-PDR1* overexpression (OE), when compared to WT, show inhibition of development of lateral branches, increased seed germination in broomrape (*P. ramose*), and darker green plants due to induction of photosynthetic pathways, all of which are hallmarks of SL signaling pathways. PIN proteins decrease in stems and roots after application of GR24. On the other hand, GR24 induces PIN2 apical and vacuolar localization. Consistent with these results, in *PDR1* overexpressing root tips, *PhPIN1* is down- and *PhPIN2* is up- regulated, which is consistent with the phenotype in *PDR1-OE* shoot. Therefore, SLs shape the pattern of its transport not only by direct induction of *PaPDR1* expression but also via auxin signaling by differential regulation of *PIN1* and *PIN2* (Sasse et al., 2015). Recently, identification of *NtPDR6*, an ABCG transporter in tobacco, suggests the existence of a common regulatory mechanism in SLs transport and signaling across plant species (Xie et al., 2015).

### Epigenetic Regulation

DNA methylation has been associated with SL signaling specifically during germination process studied in *P. ramosa* (Hemp broomrape), which requires a 4-day conditioning period for seed germination. The treatment with GR24 activates *PrCYP707A1*, an ABA catabolic gene, during germination process, reducing the level of ABA, a seed dormancy hormone. This process of activation involves DNA methylation. Treatment with 5-azacytidine (hypomethylation reagent) shortens the conditioning period. Conversely, treatment with hydroxyurea (hypermethylation reagent), inhibits *PrCYP707A1* expression and subsequently seed germination (Lechat et al., 2015). Probably GR24 causes hypomethylation of *PrCYP707A1* thereby reducing ABA levels and inducing seed germination.

## Strigolactones in Plant Stress

### Drought and Salinity

Strigolactones have been added to the growing list of plant hormones implicated in signaling pathways activated during biotic and abiotic plant stresses such as ABA, ET, JA, and SA (Xiong et al., 2002). A correlation between ABA and SL signaling during water stress is demonstrated in tomato using LC-MS/MS, enzyme specific inhibitors, and ABA deficient mutants (*notabilis*, *sitiens*, and *flacca*). Treatment of WT plants with the NCED inhibitor abamineSG and the untreated ABA deficient mutants exhibit reduced ABA and SL levels. Moreover, expression analysis in ABA deficient mutants revealed down-regulation of *LeCCD7* and *LeCCD8* transcripts (López-Ráez et al., 2010). A loss-of-function approach in *Arabidopsis* revealed a positive regulatory role for SLs in the drought stress response, supported by rescue of the germination phenotype by SL treatment in SL biosynthesis mutants (*max3* and *max4*) but not in a SL response mutant (*max2*). Moreover, SLs regulate drought stress response partially through ABA signaling, indicated by lower sensitivity of all the *max* mutants to ABA as compared to WT during germination under drought stress conditions (Ha et al., 2014). Additional evidence in *Arabidopsis* in support of ABA mediated SL response comes from increased transpiration rates and stomata density and alteration in ABA-mediated stomata closure. Microarray analysis in *max-2* and WT plants revealed a SL network in abiotic stress tolerance involving previously characterized abiotic stress responsive genes and phytohormones (ABA and CK). *max-2* mutant plants show down-regulation of ABA import genes (*ABCG22* and *ABCG40*), CK catabolism genes (*CKX1*, *CKX2*, *CKX*3, and *CKX5*), positive regulators of ABA and osmotic stress (*CIPK1*), and abiotic stress responsive genes (*AtNAC2*; Ha et al., 2014). A parallel study conducted by Bu et al. (2014), implicated *MAX2* in drought, salt, and mannitol stresses and during seed germination. Additionally, they showed that *max2* mutant plants under drought stress have thinner cuticle and larger stomata aperture. Quantitative real-time reverse transcription PCR (qRT-PCR) assays indicated that dehydration (drought stress) led to reduced expression of ABA-inducible marker genes, including *Responsive to ABA*, *RD29A* (*RESPONSIVE TO DEHYDRATION29A*), *RD29B* (*RESPONSIVE TO DEHYDRATION29B*), *COR47 (COLD-REGULATED PROTEIN47)*, and *KIN1 (COLD INDUCIBLE)* and genes involved in the ABA biosynthesis, catabolism, transport, and signaling pathways, including *NCED3*, *ABCG22*, *ABA Insensitive1* (*ABI1*), *Cytochrome P450* 707A3, and *Hypersensitive to ABA1*. Interestingly, this expression profile is *max2* specific and not observed in other SL signaling pathway genes, suggesting that *MAX2* might act as a common component of different signaling pathways, for example *MAX2* is also involved in Karrikin signaling pathway. *MAX2* expression is induced by *ABI3* and *ABI5*, two TFs acting in ABA signaling during seed germination and seedling stage, while ABA slightly down-regulates *MAX2* expression at the adult stage, suggesting that *MAX-2* acts downstream of ABA signaling (Bu et al., 2014).

Osmotic stress can be induced by drought, freezing, or salt stresses and represents a major limitation to crop productivity all over the world. Both ABA and SLs are carotenoid derivatives and given the role of ABA in drought stress, a crosstalk between SLs and ABA signaling during abiotic stress and seed germination can be speculated. Under drought conditions plants accumulate ABA, required for stomata closure (Zhu, 2002). The increase in ABA is due to increased NCED activity, catalyzing the rate-limiting step in ABA biosynthesis (Nambara and Marion-Poll, 2005). In Lotus (*L. japonicus*), osmotic stress decreases SL levels in tissues and root exudates, primarily by altering transcription of SL biosynthetic and transporter encoding genes. Pre-treatment of plants with SLs inhibited the osmotic stress-induced ABA increase in roots by down-regulating the ABA biosynthetic gene *LjNCED2*. During osmotic stress, SL levels decrease to allow an increase in ABA in the roots of lotus plant. Evidently, the SL metabolism and effects on ABA levels are opposite in roots and shoots under stress conditions (Liu et al., 2015).

### Reactive Oxygen Species

Plants produce reactive oxygen species (ROS) in various cell compartments during photosynthesis, photorespiration, electron transport in mitochondria, and biotic and abiotic stresses (Foyer and Noctor, 2005). NADPH-oxidase and apoplastic peroxidases are major sources of ROS production. ROS have emerged as major second messenger molecules acting as signals to modulate gene expression, which in turn helps in adaptation to various stresses (Sagi and Fluhr, 2006). Phytohormones are known to regulate plant development and stress adaptation by activating ROS production through NADPH oxidase encoded by *RESPIRATORY BURST OXIDASE HOMOLOG* (*RBOH*; Sagi and Fluhr, 2006). SL signaling has also been associated with ROS responses, though indirectly. The link between SLs and ROS comes from the finding that *FAR-RED ELONGATED HYPOCOTYL3* (*FHY3*) acts as a negative regulator of *RBOH* genes. *FHY3*, a transposase-related TF, is a key component of phytochromeA signaling and the circadian clock, involved in far-red (FR) light response (Lin et al., 2007). *FHY3* suppresses both root and shoot branching in *Arabidopsis fhy3max2* double mutant plants, suggesting *FHY3* acts as a suppressor of *MAX2* (Ouyang et al., 2011). It has been shown that inactivation of *FHY3* causes increased expression of *RBOH* genes, which could be responsible for suppression of branching. Moreover, *RBOH* has been shown to regulate shoot branching in tomato, *L. esculentum*, where antisense *RBOH* expression causes increased shoot branching (Koltai et al., 2011). Another link between SLs and ROS comes from the role of SLs in drought and salt stress. *max2* mutant plants show increased sensitivity to these stresses and impaired ABA response including effects on stomata closure and expression of stress responsive genes. ROS is a known second messenger during ABA signaling and it is quite likely that *RBOH* is involved in SL-dependent shoot and root branching regulation and other stress responses (Xia et al., 2015).

Strigolactones production is enhanced in response to nutrient deprivation including phosphate and nitrates, resulting in enhanced LR production. Similarly, ROS production is enhanced in response to nutrient deprivation (Shin and Schachtman, 2004). Moreover, transcriptome analysis in *M. truncatula* roots has shown that activation of NADPH oxidases under P- and N- limiting conditions results in expression of SL biosynthesis genes (Bonneau et al., 2013).

### Temperature

Seed germination in plants is subjected to optimum temperature requirement, for example seed germination in *Arabidopsis* seeds is inhibited by high temperature. Phytohormones such as ABA, CK, and GA are implicated in the seed germination process, where ABA is a negative regulator and GA and CK are positive regulators of seed germination (Miransari and Smith, 2013). SLs are known to induce seed germination not only in root parasitic weeds, but also in other plants. Germination in SL-defective *Arabidopsis* mutants under high temperature conditions is stimulated by GR24 application. Moreover, GR24 reduces the ABA to GA ratio and increases CK levels. RT-PCR analysis revealed that GR24 represses transcription of *NCED9*, an enzyme required for ABA biosynthesis (Tsuchiya et al., 2010). Similarly, SLs release *P. ramosa* (broomrape) seed dormancy by reducing ABA levels during warm stratification (Lechat et al., 2015).

### Karrikins

Karrikins are methyl-butenolide containing pyrolysis products. Karrikins are formed from burnt vegetation and function as an abiotic cue for germination in post fire habitat. A genetic screen for *karrikin-insensitive* (*kai*) mutants revealed that karrikin signaling requires *MAX2* function (Nelson et al., 2011). KAR_1_ and KAR_2_ are known germination stimulants of *Arabidopsis*, promoting germination of dormant *Landsberg erecta* seeds in addition to GR24. Both karrikin and GR24 inhibit hypocotyl elongation in WT and *max1*, *max3*, and *max4* plants during photomorphogenesis. On the other hand, this phenotype remained unaffected in *max2* mutant plants. Moreover, *MAX2* is required for induction of early transcription markers of karrikin response in *Arabidopsis* including *STH7* (*At4g39070*), a double B-box domain TF, *KUF1* (*At1g31350*), an F-box protein, and *KUOX1* (*At5g07480*), an oxidoreductase. GR24 as well as karrikins upregulate the transcript levels of all these genes. In contrast, no enhancement was observed in the *max2* mutant. Besides, karrikin also regulates *MAX4* and *IAA1* in *MAX2*-dependent manner (Nelson et al., 2011).

### Biotic Stress

Salicylic acid, JA, and ABA play major roles in plant defense responses (Robert-Seilaniantz et al., 2011). A tomato mutant, *Slccd8*, is more susceptible to pathogens including *Botrytis cinerea* and *Alternaria alternata* and shows reduced levels of JA, SA, and ABA as determined by High Performance Liquid Chromatography-Tandem Mass Spectrometery (HPLC-MS/MS). Moreover, expression of *PROTEINASE INHIBITORII* (*PINII*), a JA dependent gene and a JA response marker gene, is also repressed in this mutant. This gene has been previously shown to be involved in resistance to *B*. *cinerea* in tomato suggesting, that SLs might regulate biotic stress tolerance at transcription level (Torres-Vera et al., 2014). Using a reverse genetics approach, *MAX2* was identified as a component of plant defense response during disease resistance. *max2* mutant plants showed increased stomata conductance probably promoting pathogen entry into the apoplast and increased susceptibility to *Pseudomonas syringae* (hemibiotroph) and *Pectobacterium carotovorum* (necrotroph). Moreover, these plants show decreased tolerance to pathogen-triggered ROS and hormonal signaling (Piisilä et al., 2015). In conclusion, an SL signaling network exists in plant defense responses and this involves crosstalk with other phytohormones.

*In silico* analysis of the promoter region of the four *Arabidopsis* SL biosynthetic genes led to the identification of 19 *cis*-regulatory motifs. These motifs are present in multiple copies and majority is related to processes that were already described as being regulated by SLs. The various motifs identified include ATHB-1, GATABOX, SURECOREATSULTRII, involved in nutrient stress, GTCONSENSUS in response to light, and ACGTATERDI, MYBIAT, in response to drought stress. Others for which SL have not been functionally characterized include WBOXATNPRI and ASFMOTIFCAMV, which are involved in biotic stress. These two *cis*-elements are also required for SA signaling. Both of the above transcription elements play a role in plants defense reactions against viruses, bacteria, and fungi. Motifs like BIHD1OS and WRKY1OS were also identified in rice SL biosynthesis genes. Additionally, flooding response motif ANAERO1-3CONSENSUS was also determined in rice (D17/HTD1 and MAX1) and all the *Arabidopsis* SL genes (Marzec and Muszynska, 2015).

## Conclusion

**FIGURE 4:**
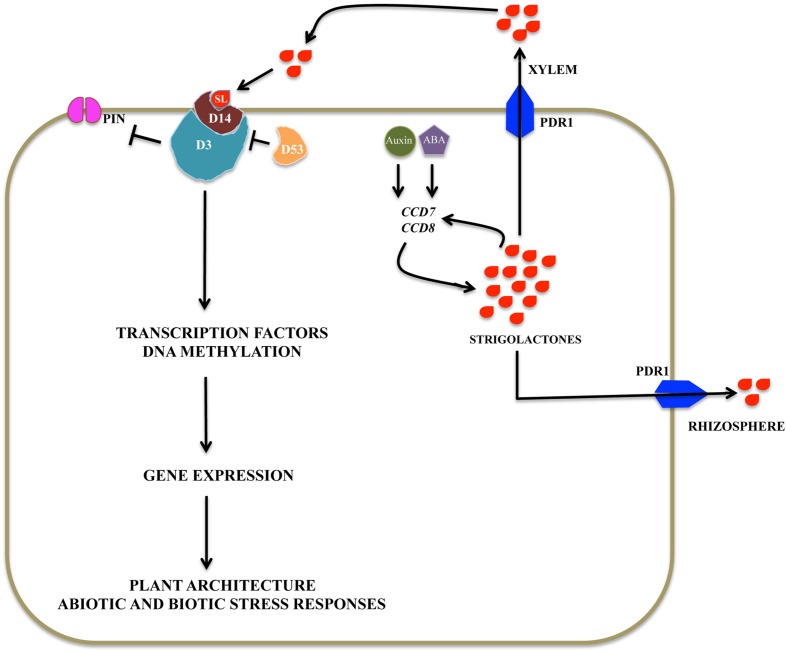
**Possible strigolactone signaling pathway: Signaling components of SLs signaling pathway in rice include a putative αβ hydrolase receptor (D14), F-box protein, a component of SCF complex (D3), and a ClpATPase (D53).** This complex regulates gene expression by controlling degradation of various transcription factors, which act as either repressors or activators of transcription. SL distribution is regulated via PDR1 transporter within the plants and outside into the rhizosphere.

Strigolactones have emerged as carotenoid-derived plant secondary metabolite molecules involved in both endogenous and exogenous signaling responses. Exogenously, SLs act as stimulants in hyphal branching during AMF symbiosis, nodulation in leguminous plants, and seed germination in parasitic weeds. Endogenously, SLs regulate shoot and root architecture, secondary growth, senescence, and fruit ripening. Both endogenous and exogenous signaling pathways are activated in response to various environmental stimuli such as light, temperature, nutrient availability, and abiotic and biotic stresses. **Figure 4** depicts the possible signaling components involved during SL signaling. SLs have emerged as an important component of signaling network comprising auxin and cytokinins in responding to various stimuli. Due to their role in developing tolerance toward various stresses SLs can serve to generate genetically modified crop plants, which can help to resolve the global food grain problem. Moreover, SL signaling pathways can be modified for horticulture applications.

A recent study has shown that the SL analogs can be used in anticancer therapy by inducing cell cycle arrest, cellular stress, and apoptosis in tumor cells. Interestingly, they had minimal effect on the growth and survival of normal cells. In the future, it will be useful to study the effects of natural SLs on cancer cells and development of SL producing plants for anti-cancer therapy (Pollock et al., 2014).

## Author Contributions

All authors listed, have made substantial, direct and intellectual contribution to the work, and approved it for publication.

## Conflict of Interest Statement

The authors declare that the research was conducted in the absence of any commercial or financial relationships that could be construed as a potential conflict of interest.
