# Corrigendum: Emerging Roles of Strigolactones in Plant Responses to Stress and Development

**DOI:** 10.3389/fpls.2016.00860

**Published:** 2016-06-24

**Authors:** Amita Pandey, Manisha Sharma, Girdhar K. Pandey

**Affiliations:** Department of Plant Molecular Biology, University of DelhiNew Delhi, India

**Keywords:** plant hormones, strigolactones, abiotic stresses, biotic stresses

In the original article, Section “Strigolactones and Plant Growth and Development,” Sub-section “Senescene,” Liu et al. (2013) should have been cited instead of Czarnecki et al. (2013).

Similarly, in the section “Strigolactone Biosynthesis,” Sub-section “Carotenoids,” last paragraph, the reference Schwartz et al. (1997) should be considered instead of Schwartz et al. (2004).

In addition, in the Section “Regulatory Mechanisms of Strigolactone Signaling,” Sub-section “Transcription,” second paragraph, the reference Nakamura et al. (2013) should not be considered for this publication.

The authors apologize for these errors. These changes do not affect the scientific conclusions of the article.

## Author Contributions

AP has contributed to the writing of this MS. MS has contributed to reading and editing of the MS. GP has contributed to the critical reading and editing of the MS.

### Conflict of interest statement

The authors declare that the research was conducted in the absence of any commercial or financial relationships that could be construed as a potential conflict of interest.
